# From Streets to Tables: Bottom–Up Co-creation Case Studies for Healthier Food Environments in Vietnam and Nigeria

**DOI:** 10.1016/j.cdnut.2024.104395

**Published:** 2024-06-29

**Authors:** Brice Even, Scarlett Crawford, Oluyemisi F Shittu, Mark Lundy, Sigrid Wertheim-Heck, Folake O Samuel, Elise F Talsma, Giulia Pastori, Huong Thi Le, Ricardo Hernandez, Inge D Brouwer, Christophe Béné

**Affiliations:** 1 International Center for Tropical Agriculture (CIAT), Hanoi, Vietnam; 2 CGIAR System Organization, Montpellier, France; 3 Department of Human Nutrition and Dietetics, University of Ibadan, Nigeria; 4 International Center for Tropical Agriculture (CIAT), Cali, Colombia; 5 Department of Global Nutrition, Division of Human Nutrition and Health, Wageningen University and Research, the Netherlands; 6 Institute for Preventive Medicine and Public Health, Hanoi Medical University, Vietnam; 7 International Food Policy Research Institute, Washington DC, United States

**Keywords:** inclusive food systems, food retail environment, healthy diets, co-creation, participatory approach, low-income populations, low- and middle-income countries

## Abstract

Current food systems fail to provide equity, sustainability, and positive health outcomes, thus underscoring the critical need for their transformation. Intervening in food environments holds substantial promise for contributing to this much-needed transformation. Despite scholars and practitioners often recognizing the necessity for bottom–up approaches, there is a dearth of empirical investigations evaluating the potential of these approaches to contribute to food system transformations in low- and middle-income countries (LMICs). Our study aimed to address this research gap, providing a unique perspective in this regard. Drawing on evidence from two co-creation case studies conducted with small-scale informal fruit and vegetable vendors and poor consumers in Vietnam and Nigeria from January 2020 to December 2021, we explored the relevance of bottom–up community-engaged co-creation processes in intervening within LMICs’ food retail environments. Employing a mixed-methods approach that includes quantitative surveys, qualitative interviews, participatory workshops, and focus group discussions, we demonstrated that bottom–up co-creation processes involving marginalized socioeconomic groups can generate retail-level innovations that are tailored to informal retail contexts, whereas remaining aligned with established top–down theories and literature pertaining to food environments and healthy diets. We provided empirical evidence highlighting how both vendors and consumers respond positively to the co-created innovations. Expanding upon our results, we offered methodological insights applicable to interventions targeted at food environments in LMICs, and considerations for future research or development initiatives in this domain. Our findings revealed the capacity of vulnerable stakeholders to actively engage in public health initiatives and contribute to developing innovative solutions that are context-specific and conducive to the adoption of healthier dietary practices. These results confirm the potential of bottom–up, co-creation, real-world interventions within informal settings to contribute toward fostering inclusive transformation of food systems.

## Introduction

Today’s food systems fall short in delivering outcomes for a healthy, equitable, and environmentally sustainable planet [[1], [2], [3]]. Global data reveal that in 2022, 149 million children under 5 years old were stunted, 45 million were wasted, and 37 million were overweight or living with obesity [4]. Concurrently, the data indicate that in 2022 ∼2.5 billion adults were overweight, including 890 million considered obese [4,5]. Every country is affected by at least one form of malnutrition [6], and food systems in low- and middle-income countries (LMICs) often simultaneously beget under- and overnutrition [[7], [8], [9], [10]]. This duality of under- and overnutrition in LMICs underscores the flaws of current food systems, which fail to provide nutritious and sustainable diets for all. Contemporary food systems often prioritize economic efficiency over nutrition, health, and equity outcomes [11]. The current decline in crop diversity constitutes a threat to food and nutrition security, reducing dietary diversity and essential micronutrient availability [6,12]. Concurrently, the prevalence of inexpensive, nutrient-poor, processed foods contributes to the spread of overnutrition and related noncommunicable diseases [2,7]. Economic disparities further exacerbate these nutritional challenges, affecting notably food affordability and accessibility, and contributing to both undernutrition and overnutrition [1,2]. Contemporary food systems are also the main drivers of other global issues including biodiversity loss [[13], [14], [15]], climate change [16,17], and growing inequities [11,18], stressing the urgent need for a “Great Food Transformation” [19].

Recent publications highlight the influence of diets and food consumption practices on food systems sustainability [[20], [21], [22], [23], [24]]. As stated by Meybeck and Gitz [25], “*diets are both the results and the drivers of food systems*,” and the role of diets, as a driver of change, deserves more attention. Recognizing the direct influence that diets have on food systems, it becomes essential to intervene at the level of food environments, which are integral parts of the food systems and refer to the interface where food consumers engage with the food system to make decisions about acquiring, preparing, and consuming food [[26], [27], [28]]. By definition, food environments encompass all aspects of the food system that affect food choices and diets. Therefore, by targeting food environments for intervention, we aimed to address critical issues within the broader food systems, thereby contributing to the necessary transformation toward food systems that are more sustainable, equitable, and capable of providing nutritious, accessible, and affordable food to all populations.

Hybrid forms of governance – where governance is defined as the structures and processes that inform, direct, manage, and monitor the activities within food systems – integrating hierarchical top–down approaches with bottom–up approaches have been widely acknowledged to be of primary importance [[29], [30], [31], [32]]. However, current approaches to transforming food systems and food environments too often rely mainly on top–down mechanisms, lacking sufficient downward accountability [28]. In contrast, bottom–up community-engaged approaches are rooted in contextual differences and cultural nuances, making them particularly suitable for accounting for and building up social differentiation and contextual complexity of food systems [28,33]. These approaches give voice and representation to marginalized communities, provide mechanisms for downward accountability, and help spur new social norms [32,34]. As a result, they can contribute to the creation of more equitable, resilient, and sustainable food systems that meet the diverse needs of all individuals and communities, while addressing the complexity of food systems. This is especially true in LMIC urban settings, where the food retail sector is often dominated by a large proportion of informal, small-scale, vulnerable actors, and frequently overlooked in top–down national or even local policy making [34].

One approach to implementing bottom–up strategies within food environments involves initiating a co-creation process, whereby local stakeholders are engaged in both the design and implementation of food environment innovations [35,36]. Such processes can be carried out through community-engaged co-creation initiatives, and, if pertaining to a research project, can be conducted through a Participatory Action Research (PAR) approach. Despite the extensive literature on co-creation [35,37], which encompasses co-design, there is a dearth of information regarding the use of co-creation processes to intervene in LMIC food environments. A recent systematic review [38] examining co-creation processes for creating healthier food environments identified 20 distinct studies, none of which took place in LMICs or involved the informal sector – characterized by economic activities conducted within unincorporated, small, or unregistered enterprises, frequently operating outside formal monetary, regulatory, and institutional arrangements [39]. The potential for capacity building and involvement of low-income and informal actors within hybridized (in)formal food environments is yet to be explored.

Drawing on evidence from two co-creation case studies conducted in Vietnam and Nigeria, this article explores the relevance of bottom–up community-engaged co-creation processes in intervening within LMICs’ food environments. Firstly, we argue that co-creation processes with low-income populations can yield retail-level innovations tailored to informal food retail sector contexts, whereas remaining aligned with established theories and literature pertaining to food environments and healthy diets; thereby allowing the integration of top–down approaches to attain hybridized forms of governance. Secondly, we demonstrate how in Vietnam and Nigeria, both vendors and consumers responded positively to the co-created innovations. This confirms the potential of bottom–up, co-creation real-world interventions within informal practices to contribute toward fostering inclusive transformation of food systems. Finally, we discuss our findings and provide methodological considerations to strengthen the implementation and contribution of bottom–up community-engaged approaches to promote inclusive food systems transformation.

## Methods

### The FVN project

This study was conducted as part of the "Fruit and Vegetable Intake in Vietnam and Nigeria" (FVN) project, which aimed to enhance the consumption of fruits and vegetables among low-income urban residents in urban and peri-urban areas of Hanoi, Vietnam, and Ibadan, Nigeria. According to the most recent income classification guidelines by the World Bank, both Vietnam and Nigeria are categorized as LMICs [40]. The project was structured around 2 distinctive components, denoted as work package 1 (WP1) and work package 2 (WP2). WP1 encompassed several preliminary “baseline” studies including a market survey, a household survey, a consumption barrier analysis survey, and a dietary intake survey. Subsequently, WP2 involved the implementation of distinct interventions within the food environment. These interventions included a client-specific coupon system (WP2b), co-created communication campaigns (WP2c), and co-created retail-level innovations with small-scale fruit and vegetable (in)formal vendors (WP2a). This article specifically concentrates on the latter component, namely, the co-created retail-level innovations (WP2a). Our research was carried out in selected low-income urban and peri-urban areas of Hanoi and Ibadan, targeting primarily informal open-air vendors who serve as the main source of food for the low-income urban population. In Hanoi, informal open-air vendors play a significant role in meeting the dietary needs of low-income individuals, accounting for ∼70% of their overall food consumption [41]. Similarly, in sub-Saharan Africa, traditional markets and informal traders serve as the primary providers of fresh foods for low- to middle-income urban and peri-urban dwellers [42,43]. The informal food retail sector, characterized by its absence of formal business registration, predominantly small-scale operations, and often lower adherence to formal economic regulations and tax obligations, is instrumental in providing fresh food to low-income urban populations in LMICs [39]. Recognizing its vital importance, the informal food retail sector appears as a key setting for deploying impactful interventions aimed at enhancing the sustainability of food systems.

### Area and participants’ selection

In both Vietnam and Nigeria, one urban setting and one peri-urban setting were chosen for project implementation. The final selection of the targeted areas was based on the high prevalence of low-income households. In Hanoi, Dong Da (urban) and Ha Dong (peri-urban) districts were selected. In Ibadan, Abaeja (urban) and Bagadaje (peri-urban) neighborhoods were selected, within the Local Government Area (LGA) of Akinyele. In 2019, the populations of Ha Dong and Dong Da districts were recorded at 397,854 and 371,606, respectively [44]. In 2020, the population of Akinyele LGA was estimated at 335,966 people [45].

The study involved a total of 175 participants, consisting of 70% vendors and 30% consumers. Vendors were identified through a market census conducted as part of the WP1 market survey. Eight marketplaces were included in the study: four in Vietnam and four in Nigeria. The selection of vendors was based on their willingness to participate in the study, active presence in the targeted markets, and their voluntary engagement in selling nutritious fruits and vegetables. Vendors were picked from the census list to build a representative sample of the different retailer types identified in the market census. In Dong Da, only informal wet/street markets were represented. In Ha Dong, formal wet markets and informal wet/street markets were represented. In Nigeria, all vendors were characterized as informal retailers, operating either directly within or in close proximity to the targeted district markets. In Vietnam, fruit vendors were prioritized over vegetable vendors due to the higher prevalence of underconsumption of fruits compared with vegetables (with a strategic exception made to include vegetable sellers in cases where an insufficient number of fruit vendors were available).

In both countries, consumers were selected (convenience sampling) from a list of poor households (1097 in Vietnam; 1255 in Nigeria) previously identified through the WP1 household survey. Participants were contacted through mobile phone communication. They were required to be the individuals responsible for food shopping on behalf of their households. Additionally, participants needed to express their willingness and availability to attend all co-creation workshops conducted as part of the study. In Vietnam, low-income households were defined as those with a monthly income below 2,650,000 VND (equivalent to USD 117 in January 2018), determined from the first- and second-income quantiles of the Vietnamese Household Living Standard Survey. In Nigeria, the local research team identified low-income households through an evaluation of housing conditions, considering factors such as roof material, fence type, and the presence of wall plaster. The sample predominantly consisted of women in both countries (99%), with only one male vendor in Hanoi. This aligned with the demographics of the shopping and vending population in the selected areas [43,46].

### Ethical approval

The ethical approval for this research was granted by the Institutional Review Boards of Hanoi Medical University (45-18/HMUIRB) and the Institute of Advanced Medical Research and Training at the University of Ibadan (UI/EC/20/053). Informed consent was obtained from all study participants, who were provided with a detailed explanation of the research aims and procedures. Data were kept anonymized and identification numbers were used for each subject. Participation was completely voluntary, and participants were informed that their anonymized data may be utilized in future analyses. Participants received financial incentives to compensate for the time dedicated to participating in the co-creation workshops, thereby mitigating potential income losses incurred from their temporary absence from vending stalls.

### Case study design

Our research methodology aligns with Yin's [47] case study approach, which is instrumental in exploring complex phenomena within their real-life contexts. This framework is especially pertinent when the phenomena and their contexts are intertwined and not easily separable, as is the case with food systems in LMICs. Our study design aligned with the five key components of case study design as outlined by Yin.

- Our central “*research question*” asked whether bottom–up, community-engaged co-creation processes hold potential to influence LMICs' food environments and food systems toward more sustainable outcomes.
- Our “*proposition*” hypothesized that bottom–up, community-engaged co-creation processes can lead to tangible changes in LMICs’ informal food retail environment, potentially steering consumers’ food choice toward more nutritious and healthy diets.
- Our “*unit of analysis*” was groups of retailers and consumers active within 8 targeted markets (see section Area and participants’ selection) in the urban food environments of Hanoi and Ibadan.
- Our “*logical structure*,” linking our proposition to data collection, and our “*criteria for interpreting findings*,” revolved around multiple metrics, including both qualitative and quantitative data. The results of the co-design phase constituted the first piece of data, which was analyzed against the backdrop of the existing literature on enhancing food environments’ sustainability (see sections Results from the co-design phase and Relevance of the co-created innovations). Semi-structured interviews with consumers and retailers, pertaining to the monitoring system of the co-production phase, assessed the satisfaction levels of retailers and consumers with the co-created innovations (see sections Results from the co-production phase and Reception of the co-created innovations). In addition, a specific module of the project endline survey explored consumers’ perceptions regarding their purchasing behavior and fruit and vegetable consumption, attributable to the co-created innovations (see sections Results from the endline survey and Effects of the co-created innovations).

More specifically, in alignment with Yin’s approach, we adopted a “*multiple case design*,” applying a consistent co-creation methodology across both Vietnam and Nigeria. This comparative set-up was particularly beneficial as it allowed for a “*cross-case synthesis*,” enabling us to identify patterns and draw conclusions applicable across the individual cases. Additionally, we adopted a “*replication logic*” where each case was treated separately to confirm or refute our hypotheses. Although this comparative approach underpins our methodological design, it is important to note that conducting participatory research in real-world settings requires adaptability and responsiveness to the unique contextual complexities and dynamics encountered in each site. Consequently, variations in context required slight variations in the methodology and tools employed in each site (see section Methodological considerations).

### Co-creation process

Our co-creation process was situated within the broader framework of PAR, which is characterized by its emphasis on inclusivity and the active involvement of participants in the research process. PAR goes beyond considering participants as subjects and instead recognizes them as collaborators, valuing their perspectives and contributions [48]. PAR, as an approach that aligns with the needed bottom–up approaches for food system transformations, typically involves the design and implementation of innovations in collaboration with local stakeholders [49,50], allowing for the exploration of context-specific changes in food environments. As a research process, by definition, PAR inherently focuses on generating knowledge through collaborative engagement with stakeholders. Contrary to the predominant focus of PAR on knowledge generation, co-creation endeavors to yield tangible outcomes and impacts. In our investigation, we adopted a PAR approach as a means of operationalizing and documenting a co-creation process. The foundational principles of participation, involvement, and engagement from PAR shaped how we approached and framed stakeholders’ relationships within our co-creation process in practice.

In the literature, the terms “co-creation,” “co-design,” and “co-production” are often used interchangeably to describe collaborative bottom–up initiatives involving multiple stakeholders [35,38,51]. The precise definitions and boundaries of each term, however, often lack clarity, which sometimes contributes to confusion and diverging perspectives among practitioners and academics. In this study, in line with Vargas et al. [38], we adopt a broad definition of co-creation that encompasses both co-design and co-production.

- Co-design refers to a process that actively engages relevant stakeholders in the collaborative design of an innovation to ensure that resulting solutions align with their specific needs [52]. Primarily conceptual in nature, co-design notably revolves around the generation of ideas, concepts, and plans.
- Co-production refers to the engagement of multiple stakeholders in the practical implementation of the co-designed innovation, with a particular focus on achieving tangible outcomes [52,53]. Co-production extends beyond conceptual realms and focus on empirical experience through real-life implementation.
- Co-creation is the sum of the co-design and co-production activities. It encompasses the collaborative ideation and planning processes (i.e., co-design), as well as the tangible execution and implementation of the co-designed innovations (i.e., co-production).

**FIGURE 1:**
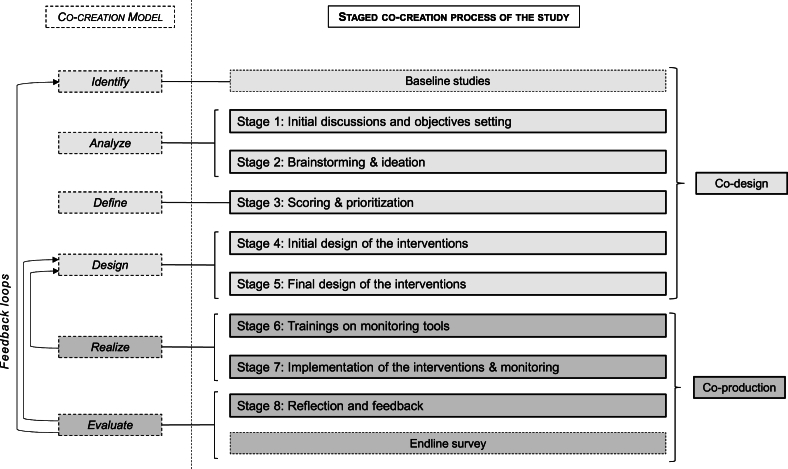
Co-creation methodology following a staged process.

Accordingly, our co-creation process consisted of 2 main phases: the design phase (i.e., co-design) and the implementation phase (i.e., co-production). Figure 1 represents our staged co-creation process and shows how it aligns with the 6-steps co-creation model proposed by de Koning et al. [54] and further developed by Vargas et al. [55]. Step 1 “*Identify*” focused on identifying the relevant structures and stakeholders that should be involved. The main emphasis here was on spotting opportunities for generating value and solutions to problems. Step 2 “*Analyze*” involved developing a comprehensive understanding of how stakeholders interact and integrate these insights to start ideating potential solutions. Step 3 “*Define*” expanded on the rights, responsibilities, and concepts previously identified, in order for participants to prioritize, map out future actions, and decide on steps forward. Step 4 “*Design*” involved designing innovations by establishing objectives, actions to reach those objectives, and evaluation procedures to monitor progress. Step 5 “*Realize*” corresponded to the implementation of the co-designed innovations. Stakeholders enacted the planned strategies while collecting data and information. This step occurred in rounds, with periodic reevaluation of the tested concepts. The refinement of innovations is crucial and was informed by both the iterative processes undertaken in the first 3 steps and the information collected during step 5. This cyclic approach ensured that innovations were continuously improved based on actual outcomes and stakeholder feedback, thereby enhancing their relevance and potential effectiveness. Step 6 “*Evaluate*” involved assessing the outcomes as anticipated, as well as reviewing the processes used previously, the learnings from co-creators, and strategies for sustaining the co-created innovations.

We conducted a series of co-creation workshops that followed a staged process [54,56] and incorporated techniques from human-centered design approaches [57,58]. In line with the definitions adopted in this article, co-creation workshops refer to both co-design workshops to conceptually design the innovations, and co-production workshops to share, discuss, and reflect on the implementation of the innovations. From a methodological perspective, we mostly relied on focus group discussions (FGD) articulated around tools developed by IDEO, a design and consulting firm renowned for its innovative approach to creating user-centered tools and solutions*.* We specifically used *IDEO field-guide to human-centered design* [58]; in particular, we used the following IDEO tools: Brainstorm; Brainstorm rules; Bundle ideas; Design principles; Create a concept; Co-creation sessions; Determine what to prototype; Rapid prototyping; Get feedback; and Integrate feedback and iterate.

**FIGURE 2:**
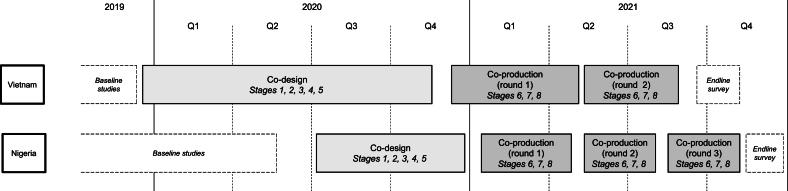
Timeline of the co-creation process in Vietnam and Nigeria. Q1 = Quarter 1; Q2 = Quarter 2; Q3 = Quarter 3; Q4 = Quarter 4.

The co-creation process extended over different durations to accommodate the specific contexts and constraints in each country. In Vietnam, the entire process spanned 19 months, beginning with a 12-mo co-design phase from December 2019 to December 2020, followed by a co-production phase lasting 7 mo, from December 2020 to July 2021. In Nigeria, the co-creation process was completed over 15 mo. The co-design phase occurred over 6 mo, from July 2020 to December 2020, and transitioned into a 9-mo co-production phase from January 2021 to October 2021 (see Figure 2).

### Co-design phase

The staged process included 8 stages. The first stage involved familiarizing the participants with issues related to dietary habits and the consumption of fruits and vegetables. Findings from prior research on local dietary habits and barriers to fruit and vegetable consumption [59] were presented to and discussed with the participants. Subsequently, participants were posed open-ended questions regarding their own eating and food purchasing habits (e.g., “*Have you consumed any fruits or vegetables in the past week?*”; “*How many servings of fruits and vegetables do you consume daily?*”), initiating an open discussion among participants and paving the way for the ideation process [60]. These initial discussions allowed to introduce and discuss the problems to be addressed, namely, the insufficient intake of fruits and vegetables and their impacts on public health and food system sustainability. Additionally, the objectives of the co-creation process were outlined, focusing on the co-design and co-production of practical and context-specific innovations aimed at increasing the purchase and consumption of fruits and vegetables, thereby improving dietary habits and the overall health outcomes of consumers.

In the second stage, participants engaged in a brainstorming session to generate ideas for increasing fruit and vegetable consumption. A series of guiding questions (e.g., “*How could vendors incentivize consumers to purchase more fruits and vegetables?”; “What could be done to make fruits and vegetables more convenient for consumers?*”) were provided to stimulate their reflections. Following group discussions, the outcomes were reviewed and deliberated upon collectively. The results of these discussions served as the basis for co-creating innovations.

Before the next stage, initial ideas from the brainstorming were clustered into groups of potential innovations by the investigators. The third stage aimed to discuss and refine these innovations and prioritize them according to the interests of vendors and consumers. Facilitators presented a set of criteria and guiding principles – including practicality of implementation, cost effectiveness, potential to increase fruit and vegetable sales, and scalability – to guide co-creators’ decision. However, it cannot be conclusively asserted that these criteria were consistently considered in the vendors’ final decision making processes. In Nigeria, vendors voted for the innovations they wished to implement. In Vietnam, the potential innovations were scored on a scale of 0–5, with higher scores indicating greater priority, and were ranked based on their perceived level of importance by co-creators. Once the innovations were ranked, the fourth stage focused on designing the innovations and developing an action plan for implementation. Participants used a template to start developing their designs, which included defining the innovations’ specific objectives, expected outcomes, and the necessary activities to achieve them. The fifth stage finalized the innovation design. In Vietnam, this stage took place a few weeks after the previous stage, and participants were provided with a written summary of the innovations discussed in the previous workshops. Vendors were grouped based on their selected innovation and worked together to finalize the design and develop an action plan outlining all the necessary steps and activities for implementation. In Nigeria, stages 3, 4, and 5 occurred within the same workshop.

### Co-production phase

In preparation for stage 6 (training on monitoring tools), the researchers developed monitoring tools to track the innovations’ implementation. One of these tools was a simplified record-keeping notebook, which was translated into Vietnamese and Yoruba. The purpose of stage 6 was to present and discuss these monitoring tools to be used during the implementation stage. During the workshops, facilitators demonstrated the use of the notebook for daily bookkeeping and provided instructions to the vendors. Vendors also had the opportunity to practice filling in sample materials and provide feedback to the facilitators. Initially, participants found the notebook to be complex and time consuming, prompting the facilitators to simplify and refine it based on their feedback. Facilitators emphasized the importance of record-keeping and monitoring, highlighting its value for both the research and the vendors' business progress over time. Despite initial resistance to engage in bookkeeping, facilitators offered support and encouragement, including the use of a Yoruba song composed by the investigators in Nigeria, which inspired participants to attempt record-keeping.

The monitoring system consisted of three tiers. “*Output indicators*” were developed to measure the effective implementation of the innovations at the vendor level on a biweekly basis. Data collection for these indicators involved field observations and discussions with vendors, supplemented by follow-up phone calls as needed. “*Performance indicators*” were developed to measure vendors’ business performance, based on purchases and sales of fruits and vegetables. Vendors themselves recorded these data using the provided notebooks (see stage 6). “*Satisfaction indicators*” were designed to gauge the perceptions of both vendors and consumers regarding the innovations, using Likert scale and open-ended questions. Data for these satisfaction indicators were collected through brief interviews with participating vendors (monthly) and random consumers (biweekly) identified as “users” of the co-created innovations; users were selected using a convenience sampling method. Monitoring data collection occurred during stage 7. “Performance” and “Satisfaction” indicators were designed to serve the measurements of the outcomes generated by the co-created innovations. However, the data from the “Performance” indicators, derived from vendors' bookkeeping, were of insufficient quality to support meaningful analysis and are therefore not presented in this article. After each implementation round (stage 7), monitoring results were compiled and utilized during reflection workshops (stage 8), to facilitate feedback loops and potentially adapt the innovations based on the findings. Descriptive statistics were used to assess the level of satisfaction of consumers and retailers toward the co-created innovations.

Stage 7 involved the practical implementation of the innovations by the vendors. This pilot stage aimed to test the co-designed innovations in a real-world context. Before the start of the implementation, a brief inception workshop was held to introduce the revised monitoring notebooks and review fundamental principles guiding the pilot stage. Facilitators collaborated closely with the vendors to execute the innovations within their specific contexts, spanning 3 implementation rounds, with each round lasting between 2 and 4 mo. In Vietnam the implementation of innovations was impacted by COVID-19 restrictions, resulting in the execution of only 2 rounds.

Stage 8 encompassed reflection workshops designed to facilitate the sharing of lived experiences and monitoring data. These workshops were conducted after each implementation round, serving as a platform for receiving feedback that would inform the refinement of innovations for subsequent rounds. Additionally, a concluding workshop took place in Nigeria to review the co-creation process, share and discuss insights on the impacts of the innovations on participants' businesses, and share lessons learned. Due to COVID-19 social-distance restrictions in Vietnam, the final reflection workshops could not happen.

After implementation took place, an endline survey was administered at the household level (through home visits) to assess the effects of the project interventions. The endline survey did not focus exclusively on our particular retail-level co-creation process but rather on the potential impact of the overall project. However, a distinct module of the questionnaire, comprising multiple choices questions, was dedicated to the co-created retail-level innovations. This article only presents the results of the aforesaid module – the overall results of the endline survey will be the object of another journal article (unpublished yet). Descriptive statistics were used to calculate the proportions of consumers who noticed the co-created retail-level innovations, purchased fruit and vegetable from the vendors implementing these innovations, and perceived an increase in their fruit and vegetable consumption in response to these innovations. These data served to measure the potential effects of the intervention on consumers’ purchasing and consumption of fruits and vegetables.

## Results

Retail-level innovations were designed and tested by local vendors and consumers and implemented in situ. Results from this co-creation process can be divided into the outputs that emerged from the co-design phase and the outcomes that emerged from the implementation phase.

### Results from the co-design phase

Nearly 50 distinct innovations were generated via the stage-2 brainstorming sessions and subsequently clustered to facilitate the design phase (see Table 1). In the “*Analyze*” step, co-creators recognized that limited food accessibility – defined by factors such as distance, time, daily mobility, and transportation modes that determine an individual’s access to food [27] – was not the primary barrier to adequate fruit and vegetable consumption, as initially hypothesized. This observation aligned with findings from the FVN baseline study (*stage 1* – see Figure 1). The initial ideas proposed by co-creators exceeded the scope of the initial research design – which focused solely on food accessibility – by encompassing a broader array of food environment domains, including food affordability, food desirability, convenience, food composition, and food outlet properties. In alignment with PAR principles, the research team decided to incorporate all co-creators' suggestions into the next stages of the co-creation process, consequently broadening the objectives and scope of the innovations to be co-designed.

**TABLE 1:** Clustering of initial ideas that emerged from the co-design brainstorming sessions in Vietnam and Nigeria

Clusters	Initial ideas (nonexhaustive)	Country
Increased assortment	⋅ Offer nutritious fruits and vegetables that are not currently sold. ⋅ Source (or produce) a wider range of seasonal and nutritious fruits and vegetables.	Vietnam and Nigeria
Nutritional information	⋅ Providing consumers with nutritional information. ⋅ Strengthen vendors’ nutrition knowledge.	Vietnam and Nigeria
Improved display	⋅ Display produce in a way that attracts more attention from consumers. ⋅ Improve hygiene practices. ⋅ Upgrade point of sale (e.g., proper stall instead of displaying produce on the floor; use of crates, baskets, pallets, etc.).	Vietnam and Nigeria
Improved user experience	⋅ Improving service quality (e.g., smile, friendly attitude, respect to poor consumers, etc.). ⋅ Provide a customer feedback system	Vietnam and Nigeria
Promotional program	⋅ “Buy 1 get 2” promotions on nutritious fruits and vegetables. ⋅ “One cheaper product everyday” for nutritious fruits and vegetables. ⋅ Offer complementary goods for purchase of nutritious products.	Vietnam and Nigeria
Online sales	⋅ Set-up a social network account and update daily with available produce. ⋅ Take orders by phone.	Vietnam and Nigeria
Home delivery	⋅ Deliver produce at consumers’ home. ⋅ Propose daily fruits and vegetables baskets delivered at home.	Vietnam and Nigeria
Deferred payments	⋅ Allow poor consumers to pay later, without interest.	Vietnam and Nigeria
Preprocessing	⋅ Prepare (e.g., cutting, milling) or process (e.g., cooking, drying, juicing) produce	Vietnam and Nigeria
Loyalty card program	⋅ Monthly cards to be ticked when consumers buy specific nutritious produce. After X number of purchases, consumers get a discount or reward.	Vietnam
Sourcing safe food	⋅ Propose a larger range of safe products. ⋅ Establish a partnership with peri-urban safe F&V producers. ⋅ Organize consumer visits to production sites/farms. ⋅ Livestream the purchasing process on the point of sales. ⋅ Communicate on food safety (e.g., “Here we have safe and traceable F&V”).	Vietnam
Addressing food safety	⋅ Use ozone generators to clean fruits and vegetables. ⋅ Clean fruits and vegetables with salty water. ⋅ Use food safety kits to test fruits and vegetables on the markets.	Vietnam
Price differentiation	⋅ Sort produce and propose different prices according to freshness and appearance. ⋅ Indicate the price of the cheaper produce so poor consumers feel more comfortable.	Vietnam
Charity actions	⋅ Giving away fruits and vegetables to the poorest.	Vietnam
Barter program	⋅ Vendors exchange their products at the end of the day (e.g., fruits against vegetables, vegetables against fish).	Vietnam
Advertising	⋅ Use of artifacts or promotional material (e.g., umbrella, hats, aprons)	Nigeria
Produce tasting	⋅ Provide consumers with sample of the products	Nigeria
Smaller bundles	⋅ Propose smaller bundles (at a lower price) of fruits and vegetables	Nigeria
Assortment bundles	⋅ Propose nutrient-rich mixed produce assortments	Nigeria
Cheaper prices	⋅ Reduce the price of nutritious produce	Nigeria

Some clusters were integrated into "composite" innovations. In Vietnam, 2 innovations were selected by vendors for implementation: a loyalty card program (VN_A) and a composite innovation that entailed nutritional information dissemination and produce display improvements (VN_B) (see Table 2). In Nigeria, 4 innovations were selected by vendors. These included upgraded product displays utilizing diverse equipment (NI_A), advertising stalls with branded accessories (NI_B), provision of nutritional information to consumers (NI_C), and the promotion of nutrient-rich mixed produce assortments (NI_D), as shown in Table 2.

**TABLE 2:** Descriptions, objectives, and food environment domains of the innovations selected and implemented by vendors in Vietnam and Nigeria

Innovations	Description	Objective	FE domain
VN_A	Vendors established a loyalty card program, offering consumers discounts upon completing 10 purchases of nutritious fruits and vegetables, with each transaction amounting to ≥50,000 VND. Upon achieving the required number of purchases, customers were rewarded with a discount on their subsequent purchase.	Incentivize customers to increase the frequency and volume of their fruit and vegetable purchases through a discount incentive program	Food affordability
VN_B	Vendors modified their stall display (e.g., using colored baskets) to emphasize the nutrient content of fruits and vegetables. In addition, vendors provided nutritional information to their consumers through verbal communication and the inclusion on their stalls of wobblers containing nutritional information.	Capture consumers’ attention and enhance awareness of the nutritional benefits of fruits and vegetables. Improve consumers’ perception of fruits and vegetables value, to increase the willingness of consumers to pay.	Food desirability
NI_A	Vendors were provided with equipment to enhance the display of their fruits and vegetables. Each participant could choose from a selection of equipment, including partitioned wooden pallets, ladders, stepwise counters, and iron vegetable hangers. The vendors were actively involved in the design process and could propose modifications to suit their individual needs.	Attract and retain customers by making the presentation of fruits and vegetables more appealing and organized. Improve consumers’ perception of the value of fruits and vegetables, to increase the willingness of consumers to pay.	Food desirability
NI_B	Vendors were supplied with promotional materials, including branded umbrellas, hats, and aprons.	Enhance the visibility of the vendors and their produce, thereby enhancing their marketability and improving the potential for sales. Umbrellas provide additional benefits, such as creating a comfortable shopping environment with shade for customers.	Food desirability Convenience
NI_C	Vendors distributed informative flyers to customers, outlining the advantages of incorporating fruits and vegetables into their diet, and providing context-specific serving sizes of select produce items to aid consumers in meeting their recommended daily intakes of fruits and vegetables.	Enhance consumers’ awareness and knowledge regarding the advantages of fruit and vegetable consumption. Foster informed decision making regarding purchases and encourage the fulfillment of recommended daily fruit and vegetable intake.	Food desirability
NI_D	Vendors proposed nutrient-rich mixed produce packs (Akapo). They also make use of communication material to promote the packs.	Offer a convenient and affordable option for consumers with time and resource constraints. Provide a diverse fruit and vegetable pack at an affordable price.	Convenience Food affordability

Abbreviations: FE domain, food environment domain; VN_A, loyalty card innovation, Vietnam; VN_B, display and nutritional information innovation, Vietnam; NI_A, display innovation, Nigeria; NI_B, advertisement innovation, Nigeria; NI_C, nutritional information innovation, Nigeria; NI_D, mixed produce packs innovation, Nigeria.

### Results from the co-production phase

The satisfaction levels of both vendors and consumers were assessed multiple times when monitoring the implementation of the innovations. Results are compiled in Table 3. The responses were overwhelmingly positive for all innovations. Key aspects raised by vendors and contributing to their satisfaction included satisfaction with the implementation of the innovations, reception of favorable feedback from consumers, and a perceived increase in the sales of fruits and vegetables. Owing to these high satisfaction levels, the characteristics of the innovations remained largely consistent across various rounds, with only minor modifications being implemented, such as changes to the design of the nutrition information wobblers. Although innovations VN_B and NI_D received the lowest scores in terms of vendor satisfaction, with 77% and 73% of vendors either satisfied or very satisfied, respectively, they were still considered satisfactory. All other innovations received a satisfaction rate of >90% among vendors. In Vietnam, vendors valued loyalty cards (91%) more highly than the improved display and nutrition advice innovation (77%). From the consumer perspective, all innovations received a satisfaction score above 90%, except for innovation VN_B, which had an 86% satisfaction rate.

**TABLE 3:** Comparative satisfaction rates for implemented innovations among vendors and consumers in Vietnam and Nigeria

	Innovations	Vendor satisfaction (%)	*n*	Consumer satisfaction (%)	*n*
Vietnam	VN_A – loyalty card program	91	22	73	125
	VN_B – display and nutritional information	77	43	83	129
Nigeria	NI_A – display	97	61	99	392
	NI_B – advertisement	98	90	96	346
	NI_C – nutritional information	97	54	97	176
	NI_D – mixed produce packs	73	20	96	41

Note: The data are from monitoring surveys conducted during the implementation stage. Respondents were asked to rate their satisfaction level on a 1–5 Likert scale (1 = very satisfied, 2 = satisfied, 3 = neutral, 4 = unsatisfied, 5 = very unsatisfied). “Vendor satisfaction” includes implementing vendors who were either very satisfied (=1) or satisfied (=2) with the innovation. “Consumer satisfaction” includes consumers who were either very satisfied (=1) or satisfied (=2) with the innovation. Consumers were selected by enumerators, using a convenience sampling method, after being observed purchasing fruits and vegetables from the implementing vendors. The number of observations (*n*) sometimes exceeds the total number of respondents because (1) satisfaction surveys were conducted multiple times during the implementation stage, and (2) some questions were aggregated to derive one single satisfaction indicator per innovation.

### Results from the endline survey

**FIGURE 3:**
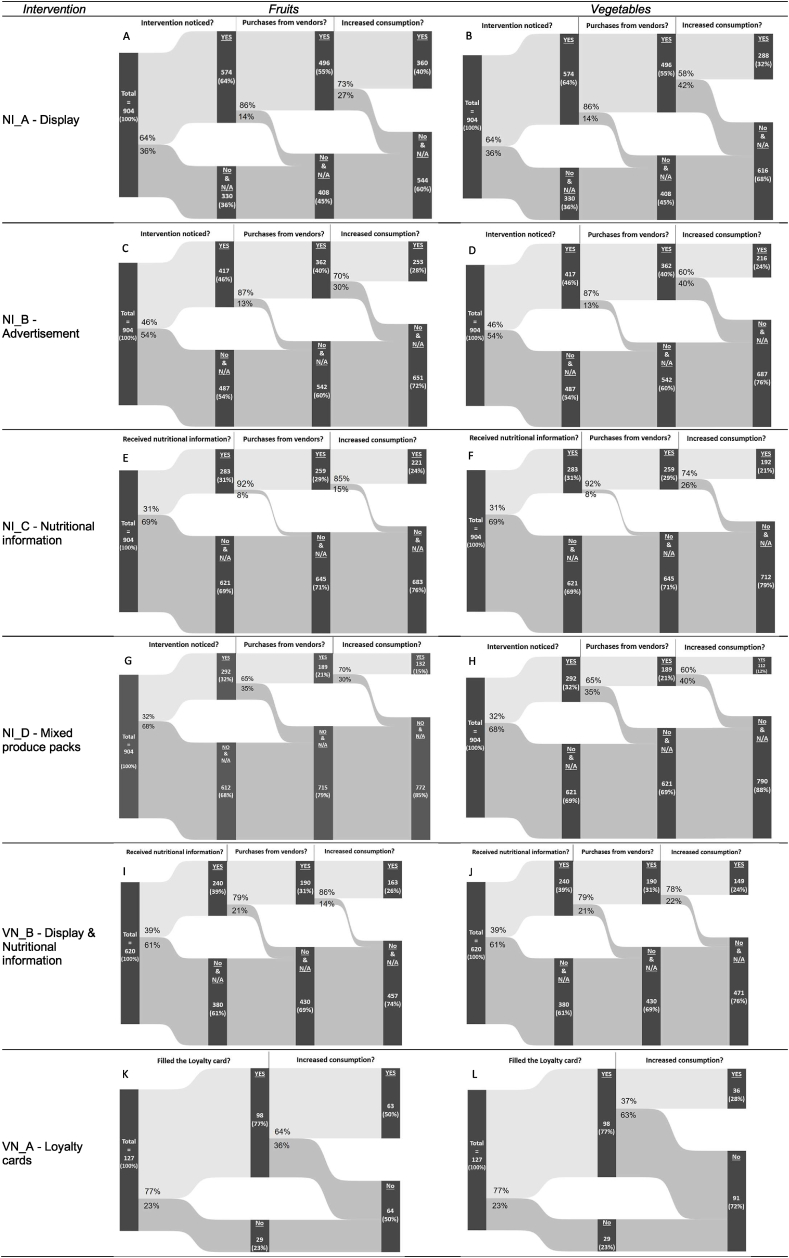
Perceived increased consumption of fruits and vegetables among consumers. Note: data were collected as part of the project endline survey, after the implementation stage ended. Samples size differed in Nigeria (904) and Vietnam (620). All respondents frequented the targeted markets and were therefore exposed to all the innovations when shopping for food; out of the 620 Vietnamese respondents, 127 received a loyalty card. “Noticed interventions?” = Have respondents noticed the co-created innovations?; “Purchases from vendors?” = Have respondents purchased fruits and vegetables from vendors implementing the co-created innovations?; “Increased consumption” = Have respondents reported an increased consumption of fruits and vegetables? VN_A = loyalty card innovation, Vietnam; VN_B = display and nutritional information innovation, Vietnam; NI_A = display innovation, Nigeria; NI_B = advertisement innovation, Nigeria; NI_C = nutritional information innovation, Nigeria; NI_D = mixed produce packs innovation, Nigeria.

The endline survey of the FVN project revealed a positive impact of our co-created innovations on consumers' perceived consumption of fruits and vegetables (see Figure 3). Participants who were exposed to the innovations and reported (1) noticing the innovations and (2) purchasing produce from participating vendors (i.e., considered as “*users*”), were asked if they noticed any changes in their fruit and vegetable consumption. Results indicated that for all innovations, over 64% of users reported a perceived increase in fruit consumption (see Figure 3). Specifically, the innovations NI_C and VN_B, which entailed the provision of nutritional information to consumers, showed the highest reported impact on fruit consumption, with >85% of users reporting a perceived increase. Although vegetable consumption also showed an increase, the results were slightly lower, with 4 innovations having >60% of users reporting an increase, and 2 innovations having 37% and 58% of users reporting an increase. Innovations NI_C and VN_B again showed the highest levels of perceived increases, with over 70% of users reporting enhanced vegetable consumption.

## Discussion

### Relevance of the co-created innovations

The co-creation process proved to be effective in designing context-specific and relevant (i.e., being appropriate and of practical value or applicability to the issue under consideration) innovations that have been shown to contribute to the conditions for healthier food environments. Drawing from existing literature, this section aims to analyze and demonstrate that, despite the lack of formal academic background among vendors and consumers involved in the process, the co-created innovations are firmly rooted in the literature on influencing food choices and fostering the development of healthy food environments.

The display and advertising innovations (VN_B; NI_A; NI_B), implemented both in Vietnam and Nigeria, aimed to enhance the desirability of nutritious food. This is in line with the literature, which suggests that strategic product placement and improved presentation increase the "food status" and can therefore increase the appeal and desirability of the promoted food [32,[61], [62], [63]]. Such interventions also modify social food environments by enhancing the attractiveness and social acceptability of food stalls, appealing to cultural factors associated with better infrastructure-equipped stalls, and ultimately increasing the legitimacy of these stalls [20]. In parallel, these interventions have also been observed to impact the consumers’ price perception and willingness to pay [64].

The nutritional information innovations that were selected for implementation (VN_B; NI_C) aligned with education interventions that aim to support food environments by providing nutritional education and food knowledge to consumers [20,65]. Point-of-purchase nutrition information, especially health messages, influences consumers' food choices and improves their perceptions of produce [64]. Such interventions address desirability by improving the social status and acceptability of promoted produce [61]. Moreover, providing nutrition advice on-site positively impacts the service quality of a store, which, in turn, has the potential to positively impact the store's price image, appealing to consumers through improved services and overall positive store perception [64], potentially leading to increased purchases of produce, fruits and vegetables in our case studies.

The loyalty card innovation tested in Vietnam (VN_A) aimed to enhance food affordability by providing financial incentives. Public health experts acknowledge the effectiveness of financial incentives and price manipulation in promoting healthy dietary choices [[66], [67], [68]]. Previous research indicates that reducing prices is among the most effective strategies to increase consumption of nutrient-dense nonstaple foods [[68], [69], [70]]. Membership programs such as a loyalty card system, offer incentives for specific targeted food items and foster repeat consumption [71]. As conditional financial incentives, wherein consumers earn the entitlement to a reward if their purchases meet a specific threshold, these programs are linked to consumer performance and effective purchases. Although this form of price manipulation is indirect, research has shown its effectiveness in increasing the purchase of healthy foods [[72], [73], [74]].

The mixed produce innovation (NI_D) aimed to improve food desirability and affordability by offering nutrient-rich mixed packages composed of several nutritious fruits and vegetables. These types of packages influence the physical food environment by enhancing convenience, offering a selection of desirable produce that can be easily purchased at once [61,75,76]. These packages also improve the economic food environment by offering value for money [20] as the price per kilogram of produce in the packages is slightly lower compared with purchasing them separately. However, concerns arise regarding the affordability of these packages for low-income consumers, as the larger volume requires a greater upfront expenditure. This may help explain the relatively lower success of this innovation compared with others (see the next section).

### Reception of the co-created innovations

Compared with top–down approaches, co-creation strategies have been found to be more closely aligned with local contexts and the needs of end-users, and often result in a greater sense of community ownership [77,78]. To evaluate the extent to which our co-created innovations were successful in achieving these outcomes, participants’ perceptions of the innovations were assessed during their implementation via quick interviews with vendors and consumers [79] to gauge their satisfaction levels. Our results indicate that the co-created innovations were overwhelmingly well received by both vendors and consumers, indicating a strong potential for adoption and success. Differences in satisfaction levels could be attributed to vendors’ perceived direct benefits, and the associated economic outcomes. The minimum purchase requirements of the cards have easily measurable impacts on sales, leading to unplanned purchases, and anchor consumers to specific vendors, increasing the likelihood of repeat purchases [64]. Additionally, loyalty cards might have been perceived as more attractive by vendors in terms of time investment. Although card distribution and development require upfront effort, they entail lower follow-up effort (apart from signing the cards after eligible purchases). In contrast, providing nutrition advice requires a more constant stream of effort over time, with vendors needing to mobilize knowledge and dedicate time to converse with consumers. In Nigeria, all innovations were received with satisfaction levels ≥ 90%, except for the mixed packs, which were associated with low sales. The possible reason for this apparent underperformance is likely to be multifaceted. Firstly, consumers' purchasing behavior in Nigeria is largely influenced by habits [79], which suggests that the novelty of the mixed packs may have hindered their acceptance by consumers. Secondly, consumers may have been apprehensive that vendors could exploit the mixed packs to conceal spoiled fruits or vegetables [80]. Additionally, the novelty of mixed packs may have led to lower vendor satisfaction rates as they perceived novel innovations as less likely to succeed compared with existing practices [81].

It seems that innovations requiring lower effort from vendors, having direct and easily observable benefits (e.g., generating immediate economic outcomes), and that are not too far from the existing retailing norms (i.e., progressive) can help bolster ownership and success [81,82]. Aligning with these criteria when designing and selecting innovations for implementation, may help promote a longer-term adoption of retail-level innovations, as well as contribute to their scalability, because other vendors in proximity may be more likely to observe the benefits of the innovations and replicate them [82].

Lastly, it is noteworthy to emphasize consumer satisfaction, as it consistently exceeded 75% across all innovations in both countries. This high level of consumer satisfaction can yield tangible benefits for vendors [83,84] and can serve as a useful indicator of the effectiveness and acceptance of the innovations. High levels of consumer satisfaction can foster adoption of the innovations by participating vendors [85], and replication by peers, and as such ultimately contribute to the creation of food environments that are conducive to healthy dietary behaviors.

### Effects of the co-created innovations

The research design did not include participants’ randomization and the establishment of a control group, thus hindering our ability to rigorously assess the impact of the co-created innovations. The endline survey results (Figure 3) revealed an apparent influence of the project intervention on the purchase and consumption of fruits and vegetables among a significant proportion of consumers. Although we cannot attribute these increases in purchase and consumption with certainty to our project, we can assume that at least part of those changes was due to the project intervention. It is also possible that the observed positive changes reported by participants may be partly attributed to their awareness of their involvement in the intervention (i.e., Hawthorne effect) [86]. Although acknowledging that strict causality cannot be demonstrated, the consumers’ self-reported increased consumption of fruits and vegetables suggests positive behavioral outcomes. This increase can also serve as a potential proxy of dietary intake and, consequently, as an indicator of positive health outcomes [87]. The evaluation of the overall FVN project [Giulia Pastori, Elise F. Talsma, Edith J.M. Feskens, Le Thi Huong, Folake O. Samuel, Oluyemisi F. Shittu, Toluwalope E. Eyinla, Alan de Brauw, Kate Ambler, Sigrid Wertheim-Heck, Ricardo Hernandez, Brice Even, Gennifer Meldrum, Amanda de Filippo, Thi Thanh Le Xuan, Ngo Thi Ha Phuong, Truong Tuyet Mai, Mark Lundy, Inge D. Brouwer, unpublished results, 2024] corroborates our results – at least partially. In their analysis, the authors found that the consumption of fruits and vegetables (considered together, as well as independently) in Nigeria, was higher among the treatment group (population exposed to the interventions) as opposed to the control group (population living in another neighborhood and therefore not exposed to the interventions). In Vietnam, however, only the intake of fruit was found statistically higher compared with the control group, not that of vegetables or of total fruits and vegetables together.

In both locations, the nutritional information innovations showed the greatest potential to create impact. The larger perceived impact of this specific innovation might be due to several factors. Firstly, nutrition advice may drive a greater impetus to change consumption because it increases consumers’ understanding of the importance of eating fruits and vegetables. This enhanced awareness, as an intermediate cognitive outcome, can ultimately lead to behavioral and health outcomes if it translates into increased consumption of fruits and vegetables. This appeals to Vietnamese consumers who prioritize food safety and nutrition over price [88] and Nigerian consumers whose knowledge of the health benefits of fruits and vegetables was observed to be a primary determinant of consumption [89,90]. Secondly, the informal conversations through which nutrition information was transmitted may have fostered trustful interactions between vendors and consumers, cultivating a sense of community and enhancing the innovation's positive perception [82]. This, in turn, could have influenced consumers to increase their purchases from knowledgeable vendors who are more adept at providing nutrition information [80], thereby nudging food choices toward a greater emphasis on fruits and vegetables.

### Contextual challenges related to the COVID-19 pandemic

The COVID-19 pandemic affected the co-design and co-production phases, causing disruptions and delays in data collection and implementation, altering interactions between facilitators and participants, and disrupting market operations. Pandemic-related restrictions necessitated adaptations in vendors' practices and approaches to sustain their activities, resulting in significant impacts on their sales. Concurrently, consumers encountered challenges related to market accessibility and adjusted their purchasing behavior due to mobility constraints. In Ibadan, Nigeria, the restrictions had adverse consequences for vendors, including reduced customer traffic, movement limitations, and disruptions in financial safety nets, as indicated by a decline in remittances received by vendors [91]. Similarly, in Vietnam, social distancing measures and restrictions on gatherings prevented informal vendors from conducting their trade, leading them to explore alternative approaches such as mobile vending or online selling, or in some cases, ceasing retailing activities altogether [92]. In Vietnam specifically, the number of implementation rounds was reduced to 2 due to the constraints on selling and movement imposed by the pandemic.

The COVID-19 pandemic serves as a stark reminder of how external dynamics can significantly impact the implementation of research or development interventions. Beyond pandemics, other external dynamics, such as natural disasters and geopolitical conflicts, can also pose challenges and disrupt intended processes and outcomes. This highlights the necessity of adopting malleable and flexible approaches that can adapt to specific contextual dynamics and ensure inclusivity in the face of unforeseen circumstances. The inherent flexibility of our participatory approach allowed us to promptly respond and adjust to changing circumstances. This ensured that the perspectives of marginalized communities were duly considered and enabled the co-creation process to persist and thrive despite external challenges. By acknowledging and addressing the influence of external factors through the use of flexible participatory approaches, researchers and practitioners can navigate the complex and dynamic nature of food environments, ultimately contributing to more resilient development outcomes.

### Methodological considerations

Our study offers some methodological insights that can help better design and implement bottom–up co-creation initiatives in LMICs’ food environments. In LMIC contexts where complex food systems present unique challenges for providing healthy and sustainable diets [27,[93], [94], [95]], and where data are often scarce, needs assessments are crucial for project design contextual relevance [96,97]. These needs assessment can be done through formalized mechanisms (such as desk research or quantitative data collection) and more informal mechanisms (such as consultation or conversations with local stakeholders). Beyond reviewing the existing literature to shape the research project and identify research sites, the FVN project conducted several preliminary studies (e.g., 24-hour recall, household surveys, barrier analysis, seasonal food availability analysis, market-level assessment) to characterize the local food environments and inform the later stages of the project (i.e., food environment-level interventions). Although the initial design of the project planned for the co-created retail-level innovations to focus specifically on accessibility of fruits and vegetables, the preliminary studies revealed that accessibility was not a major constraint for fruit and vegetable consumption. This was later confirmed by the initial discussions and brainstorming with vendors and consumers. Consequently, the scope of the co-created innovations was expanded to encompass other dimensions, such as food affordability, desirability, and convenience, rather than solely concentrating on food accessibility. This reorientation of the co-creation process was made possible by the comprehensive needs assessments conducted, underscoring the importance of robust research before engaging in any co-creation processes.

Previous instances of co-creation have been based on a deeper level of involvement of local stakeholders, beginning from the project design phase [98,99]. This engagement facilitated the collection of valuable inputs pertaining to research design and co-creation mechanisms, fostering equity, empowering participants, and acknowledging the distinct contexts and needs of the stakeholders involved. In contrast, expert-led project design (as in our case studies) may provide certain advantages, such as increased control over project implementation and the ability to meet donor requirements and timelines but may inadvertently overlook opportunities for participant empowerment and the recognition of their valuable knowledge. Adopting a flexible design approach, where experts propose mechanisms and participants provide quick feedback, could accommodate both perspectives and needs. In the context of informal retailing settings, allowing vendors and consumers to provide feedback on the project design and the proposed participatory mechanisms may serve as an ideal compromise, particularly considering the time constraints faced by vendors (e.g., in Vietnam, participants' average working time was 6.7 days/week with 9.5 active hours/day).

Co-creation case experiments commonly involve convening stakeholders in group settings [38]. Group-based co-creation offers several benefits, including community empowerment, time efficiency, and the establishment of trust among participants [[100], [101], [102], [103], [104]] and is more effective than individual engagement in generating support and enthusiasm for research participation [105]. However, group meetings have potential drawbacks such as stifling innovation, limited flexibility for individual participants regarding time and location, and the dominance of vocal individuals [106,107]. To overcome these limitations, the incorporation of breakout groups alongside plenary discussions and follow-up one-on-one discussions, as used in our research, can increase freedom of expression, manage groupthink to a certain extent, and cater to individual's needs [77,108]. These practices are particularly critical in retailing contexts, as vendors engage in co-creating innovations while still competing with each other, potentially leading to the presence of individual ideas that vendors may be reluctant to share within a larger group. Diversifying participant engagement formats (e.g., pairs, small groups), or mixing participants from multiple locations, genders, or ethnic groups can help address these challenges [109]. Establishing specific groups, for example, tailored for women or marginalized individuals [77,99], or separating elderly and youth participants, who tend to contribute less than adult participants [77] may help creating for the former a safer co-creation space. Genuine collaborations between retailers and consumers throughout nutrition intervention remains uncommon [110] and the integration of both parties into a co-creation process may pose challenges.

Our research design, and in particular our participants’ selection methods, impeded our ability to rigorously assess the impact of the intervention. The research was initially intended to use participant-collected business data (i.e., *performance indicators*) to measure innovations’ impacts on fruit and vegetable purchases. However, participant vendors were selected based on their motivation to create and implement retail-level innovations, and we did not proceed to participant randomization and the establishment of a control group (i.e., vendors collecting and sharing their business data without implementing any co-created innovations). This lack of a control group prevented the use of vendors’ data in attributing changes in sales to the intervention. Avenues could be considered to combine participatory approaches, randomization, and self-collection of data by participants [111]. This is particularly challenging, however, in informal retail settings, where informal vendors might be reluctant to collect data without the incentives and potential tangible benefits of being part of the innovation’s implementation.

The participants' profile plays a crucial role in the design and implementation of such processes. Despite the intention of applying the same methodology in both countries for the sake of comparability, the variations in participants' profiles influenced the dynamics of co-design and implementation in each country. The differences in participants' abilities to envision innovations (which might be a proxy of their education levels) influenced the tools employed in the co-creation process. Co-creation participants' aptitude for generating and evaluating conceptual solutions is often associated with factors such as low or limited literacy and numeracy, and limited design knowledge and experience [112]. In Vietnam, all vendors were literate, with 27% having completed high school or attained a higher education level [113], whereas in Nigeria, the majority of vendors lacked formal education, with limited literacy and numeracy skills [114]. These differences necessitated distinct facilitation approach and practices in each country. The Nigerian team made additional efforts to enhance participants' capacities for co-creating innovations. Facilitators employed storytelling communication methods to stimulate participants' ideas regarding potential food environment issues and solutions. In Nigeria, each co-design workshop systematically integrated supplementary training sessions to enhance participants' knowledge on various concepts, including nutrition, food safety, and customer service. In addition, some Nigerian vendors with low literacy level received extra support for bookkeeping and monitoring activities. Furthermore, our observations also suggest that variations in participants' propensity for risk-taking behavior, which may have been influenced by their income levels as evidenced by previous research [115], had an effect on their willingness to engage with the most pioneering innovations. Vietnamese participants displayed a greater inclination to adopt novel innovations compared with Nigeria.

Timeframe is also a critical design consideration, and a longer timeframe would have benefited the research. The 2-year timeframe allowed by the project limited trust building with participants, reduced the number of possible implementation iterations, and constrained the possibility of capturing seasonal effects or smoothing data noise introduced by the COVID-19 pandemic and its effects. Choosing a timeframe that allows participants to expand their capabilities, to strengthen their relationships and trust [116,117], and to critically analyze their situation and the changes induced by the innovations [109], is crucial in LMIC settings where participants often have limited education and low risk-taking profiles.

### Future research

In the pursuit of healthy and sustainable food system transformations, bottom–up co-creation approaches have emerged as promising avenues for contributing to positive change. However, understanding the impact of such initiatives is necessary to position them as effective drivers of food system transformation. Comprehensive evaluation of the co-creation process and rigorous assessment of the long-term impact of the co-created innovations are imperative to gauge their efficacy in improving the food retail environment and contributing to sustainable food system transformation. One critical aspect deserving further attention is the examination of whether the co-created innovations influence consumer perceptions, preferences, and behaviors toward sustainable and healthy food choices. Another aspect is investigating whether co-creation leads to the development of sustainable business models benefiting small-scale and informal vendors and contributes to economic viability while advancing sustainable practices. Additionally, research exploring strategies for scaling up and replicating successful bottom–up co-creation initiatives would help to maximize their potential reach and impact.

Future research should also investigate effective strategies to ensure both inclusive participation of low-income groups, and collaboration across stakeholder groups throughout the co-creation process. To sustain co-creation approaches, we need to rely on and reflect the great diversity of public, private, and civil society actors involved in the local, national, and international governance of food systems. By understanding how to effectively engage and involve a wider range of stakeholders, co-creation initiatives could also tap into a broader range of expertise and resources, potentially leading to more holistic and cohesive innovations. An essential aspect that warrants examination is the relationship between bottom–up co-creation and existing food system policies and governance structures. Identifying potential barriers and enablers in this regard is crucial, as combining bottom–up and top–down approaches can help crafting adaptive policies that complement and support community-driven initiatives and strengthen the governance of food systems. Understanding how co-creation initiatives can build a sense of mutual ownership and agency within communities and across stakeholder groups would also enhance the chance of success of such interventions. Finally, exploring mechanisms to facilitate knowledge sharing and capacity building among stakeholders engaged in co-creation initiatives could foster a collaborative learning environment, leading to more informed and impactful co-creation processes.

## Conclusion

In conclusion, our study demonstrates how bottom–up co-creation of retail-level innovations in food environments has the potential to contribute to changes in food consumption among low-income populations in Vietnam and Nigeria. By focusing on specific co-created innovations within the context of LMICs, we address a knowledge gap that exists in this area. It is important to note that these interventions are specifically targeted toward lower-income populations, heavily reliant on informal food systems under precarious conditions, necessitating cautious consideration of the potential risks posed to livelihoods.

We have observed that small-scale food vendors were willing to actively engage in co-designing innovative approaches, resulting in innovations that aligned with improving public health and nutrition. In addition, although our initial findings suggest potential gains in socioeconomic and inclusion goals, further research is needed to better assess and substantiate these claims. Despite the significant challenges, including the COVID-19 crisis, small-scale food vendors have demonstrated their ability to implement these innovations. Supporting and empowering small-scale and informal food vendors therefore emerges as an effective strategy to facilitate inclusive transformations that strive for greater equity and improved nutrition.

While our findings are promising, it is essential to refrain from making sweeping claims regarding the transformative impact of these interventions on food systems. Rather, we should emphasize the specific nature of co-creation interventions within LMIC food environments. Acknowledging the nuances and limitations of our study, our research on “bottom–up, co-creation, real-world interventions in informal settings” offers a nuanced perspective on the potential impact of such approaches within hybridized (in)formal food systems, which can lead to positive changes in food consumption, nutrition, equity, and overall public health in LMICs.

## Author contributions

The authors’ responsibilities were as follows – IDB, ML, SW-H: designed research; BE, ET, FOS, HTL, ML, OFS, SW-H: conducted research; ET, FOS, GP, HTL, OFS, RH, SC: analyzed data; BE, CB, IDB, SC, SW-H: wrote the article; BE: had primary responsibility for final content; and all authors have read and approved the final version of the manuscript.

## Conflict of interest

All authors have no conflict of interest.

## Funding

The Fruit and Vegetable Intake in Vietnam and Nigeria (FVN) project was funded by the Bill and Melinda Gates Foundation and the UK Foreign, Commonwealth & Development Office, project No. OPP1182727. The project also received support from the CGIAR Research Program on Agriculture for Nutrition and Health (A4NH) and the CGIAR Initiative Sustainable Healthy Diets through Food System Transformation (SHiFT). The funders had no role in the design of the study; in the collection, analyses, or interpretation of data; in the writing of the manuscript, or in the decision to publish the results.

## Data availability

The data described in the manuscript will be available upon request, pending the proper citation of our work in any subsequent use.
